# Self-Reported Rapid Eye Movement Sleep Behavior Disturbance and Its Associated Factors among Medicine and Health Science Students at the University of Gondar

**DOI:** 10.1155/2020/1810836

**Published:** 2020-05-15

**Authors:** Baye Dagnew, Henok Dagne, Zewudu Andualem

**Affiliations:** ^1^Department of Human Physiology, School of Medicine, University of Gondar, P. O. Box 196, Gondar, Ethiopia; ^2^Department of Environmental and Occupational Health and Safety, Institute of Public Health, University of Gondar, P. O. Box 196, Gondar, Ethiopia

## Abstract

**Introduction:**

Rapid eye movement sleep behavior disorder (RBD) is characterized by dream-enacting behavior (shouting, punching, and falling out of bed) related to unpleasant dreams and loss of normal rapid eye movement (REM) sleep muscle atonia. Rapid eye movement sleep enhances learning and memory by regulating neuronal synapses, and if it is undesirable, it can lead to cognitive impairment and poor academic performance and may end up with death. To the best of our searching databases, there is no such study conducted in Ethiopia. Therefore, this study is aimed at determining self-reported symptoms of RBD and its associated factors among the University of Gondar medicine and health science students (2019).

**Methods:**

We conducted a cross-sectional study from June 1 to July 2019, among medicine and health science students at the University of Gondar using a simple random sampling technique. A self-reported RBD screening questionnaire was used to collect the data. We used Epi Info™ 7.0.8.3 and Stata 14 for data entry and statistical analyses, respectively. Descriptive statistics (frequency with percent and mean with standard deviation) and adjusted odds ratio (AOR) with 95% uncertainty interval (UI) were computed. In a multivariable binary logistic regression, variables with a *p* < 0.05 were declared as significant.

**Results:**

Three hundred and eighty-seven students took part in the study. The mean age of participants was 20.81 (±1.83) years. The prevalence of self-reported RBD was 46.25% [95% UI (41.26%-51.24)]. Physical exercise immediately before sleep (AOR = 2.50, 95% UI (1.24-5.02)), using Facebook immediately before sleep (AOR = 1.93, 95% UI (1.18-3.15)), having daytime sleepiness (AOR = 1.92, 95% UI (1.16-3.19)), and self-reported depressive symptoms (AOR = 2.40, 95% UI (1.45-3.99)) were significantly associated with self-reported RBD.

**Conclusion:**

The current study revealed a high prevalence of self-reported RBD. This remarkable problem suggested a need to design strategies to prevent RBD symptoms among university students through targeting screening of depression, daytime sleepiness, and adjusting bedtime routines such as physical exercise and internet use immediately before going to bed.

## 1. Introduction

Sleep is a reversible loss of consciousness due to fluctuations of neurotransmitters and hormones within a 24-hour period and can exist in three states, namely, wakefulness, nonrapid eye movement sleep, and rapid eye movement (REM) sleep [1]. Rapid eye movement sleep facilitates learning and memory by regulating neuroplasticity [2]. However, abnormal REM sleep leads to REM sleep behavior disorder (RBD) [3] with common symptoms of RBD such as dream-enacting behavior (shouting, punching, and falling out of bed) related to unpleasant dreams and loss of normal REM sleep muscle atonia [4, 5]. Increased electromyography activity during REM sleep is partly linked to the nigrostriatal dopamine system, and hence, RBD is associated with dopamine function [6]. Evidences showed RBD can cause cognitive impairment [7], somatosensory impairment [8], autonomic dysfunction [9], and olfactory dysfunction [10] and reduced academic performance [11]. If untreated earlier, it could end up with death, with an evidence of 21% of mortality in a mean follow-up period of 7.1 years [12].

Prevalence of RBD is more pronounced in individuals with underlying diseases like Parkinson's disease [13, 14] and those on antidepressants [15]. However, RBD can affect all population regardless of sex and age (usually higher in elderly) [16] and those without neuropsychiatric problems [17–20]. As sleep is circumstance-dependent and students sleep inadequately due to the highly competitive and demanding learning environment [21], delayed sleep phase syndrome and insomnia are commonly observed among university students than the general population which contributes to the development of RBD symptoms [22, 23]. In North Carolina, 27% college students experienced at least one form of sleep disorder [24]; in Germany 25.9%, university students have frequent awakenings at night [25]; in the University of L'Aquila, in Italy, 8.3% nursing students have disrupted sleep and 7.7% students suffer early morning awakening [26]. Different factors are known to affect sleep quality such as sex (more pronounced in females) [11], depression [27] and living circumstances [21]. The recognition of at-risk individuals for neurodegenerative disorders may ultimately provide a platform for designing preventive strategies and treatment options [28].

To the best of our knowledge, there is no study conducted in Ethiopia to assess symptoms of RBD among university students. Therefore, the current study is aimed at determining the prevalence of self-reported RBD and identifying associated factors among University of Gondar medicine and health science students.

## 2. Materials and Methods

### 2.1. Study Setting, Period, and Population

We used institution-based cross-sectional study design at the University of Gondar, Northwest Ethiopia, from June 1 to July 10/2019. The University of Gondar students who were attending medicine and health sciences in the 2019 academic were the source population for this study. We included those medicine and health science students who were found at the time of data collection. However, we excluded students who had severe illness at the time of data collection.

### 2.2. Sample Size Determination and Sampling Technique

The sample size (*n*) was determined using a single-population proportion formula with the following assumptions: *P* (self‐reported prevalence of RBD symptoms) = 50% (since there was no such study previously in the study area), 95% UI, margin of error (*d*) = 5%, and nonresponse = 5%, *z* = the standard normal tabulated value, and *α* = level of significance. After adding a nonresponse rate of 5%, the final sample size was 404. We used a lottery method simple random sampling technique for the selection of participants for the study. The active numbers of medicine and health science students in the year 2019 were 3544. Thus, 1416 were medicine students and 2128 were health science students in different departments. We used proportional allocation for each field of study (medicine and health science students) and in each department and batch to get the required sample.

### 2.3. Data Collection Instrument and Procedure

We used a self-administered semistructured questionnaire to collect the data. The questionnaire comprised items related to sociodemographic characteristics, lifestyle (including bed routine activities), RBD screening, depression, stress, and excessive daytime sleepiness. A rapid eye movement sleep behavior disorder screening questionnaire was used to collect data related to self-reported RBD [29]. The main purpose of the RBD screening tool is to screen individuals for RBD, which could represent an early clinical manifestation of neurodegenerative diseases. This 10-item questionnaire is comprised of simple “yes” or “no” options with a total score of 13 (in that questionnaire, 6 questions are comprised of 4 subquestions). The test characteristics of the instrument are sensitivity of 96% and specificity of 56%, respectively. Beck's depression inventory (BDI-II) [30], perceived stress scale [31], and Epworth daytime sleepiness screening tool [32] were used to determine the levels of self-reported depression, stress, and daytime sleepiness, respectively.

### 2.4. Study Variables

The dependent variable includes self-reported RBD symptoms (dichotomized).

The independent variables include sociodemographic variables (age in years, monthly income, ethnicity, religion, and year of study), lifestyle and bed routine activities (Khat chewing, cigarette smoking, alcohol drinking, and coffee intake), self-reported stress, depressive symptoms, and daytime sleepiness.

### 2.5. Operational/Term Definitions

#### 2.5.1. Self-Reported Rapid Eye Movement Sleep Behavior Disturbance

In this study, we considered a person having self-reported RBD when he/she scored 5 and above of the total (13) screening items [29].

#### 2.5.2. Depression

We used the second edition of Beck's depression inventory (BDI-II) revised in 1996. When a study participant scored 21 and above of the total (63) scores of BDI-II, he/she had self-reported depression [33].

#### 2.5.3. Stress

We considered a person having stress when he/she scored 5 and above of the total (40 scores) of the 10 item questions of perceived stress scale (PSS-10) [31].

#### 2.5.4. Excessive Daytime Sleepiness

We asked 8 items, each with 3 alternatives, to assess daytime sleepiness. We categorized a person as having excessive daytime sleepiness when he/she scored 11 and above from the total score of 24 [34].

### 2.6. Statistical Analysis

After checking completeness and consistency of the collected data, the data entry clerk entered each data into Epi-info™ 7.0.8.3 then exported them into Stata 14 for statistical analysis. Frequency with percent and mean with standard deviation were computed to express descriptive results. The one-to-one binary logistic regression was performed to determine the crude association between each independent variable and self-reported RBD. Variables in the bivariable analysis with a *p* value < 0.2 were candidates for multivariable binary logistic regression analysis. From the multivariable analysis, independent variables with a *p* value < 0.05 were considered associated factors for self-reported RBD.

### 2.7. Data Quality Management

In this study, we adopted a validated questionnaire [29] for the assessment of RBD symptoms. The investigators recruited and gave orientation to 3 MSc students of Human Physiology to facilitate the data collection process. The orientation included ideas related to the objectives of the study and ethical issues during the process of questionnaire distribution and collection.

## 3. Results

### 3.1. Sociodemographic Chronicles of Study Participants

Three hundred and eighty-seven students took part in the study with 95.8% response rate. The mean age of participants was 20.81 (±1.83, range: 18-34) years. Of the respondents, 271 (70.03%) were males, 238 (61.50%) were second year and above, and 306 (79.07%) were health science students (Table 1).

### 3.2. Prevalence of Self-Reported RBD Symptoms

Forty-eight (12.40%) students experienced bruxism. Regarding bedtime routines, 11.37% consumed coffee, 6.72% drunk alcohol, 6.46% smoked cigarettes, 16.54% had physical exercise, 32.82% took a bath, and 61.76% were using Facebook immediately before going to sleep. One hundred and seventy-nine (46.25%, 95% UI (41.26%-51.24)) students reported symptoms of RBD. Of the total participants, 31.07% of students had experienced excessive daytime sleepiness, 34.73% had self-reported depressive symptoms, and 81.25% students reported perceived stress (Table 2).

### 3.3. Associated Factors of Self-Reported RBD Symptoms

We tested all independent variables for crude association with self-reported RBD symptoms using binary logistic regression. The presence of romantic relationship, preuniversity residence, bruxism, coffee consumption, alcohol drinking, cigarette smoking, physical exercise, taking a bath, using Facebook, age in years, pocket money, excessive daytime sleepiness, depressive symptoms, perceived stress, and field of study were candidates for multivariable binary logistic regression. After running multivariable analysis, physical exercise immediately before sleep, using Facebook immediately before sleep, excessive daytime sleepiness, and depressive symptoms were significantly associated with self-reported RBD symptoms. The odds of self-reported RBD symptoms was 2.5 times (AOR = 2.50, 95% UI (1.24-5.02)) higher in those who experienced physical exercise immediately before sleep than those who did not exercise. Students who used Facebook immediately before sleep were 1.93 times (AOR = 1.93, 95% UI (1.18-3.15)) more likely to get RBD symptoms than their counterparts. The odds of having RBD symptoms was 1.92 times (AOR = 1.92, 95% UI (1.16-3.19)) higher in those who had excessive daytime sleepiness than those without excessive daytime sleepiness. Students who reported depressive symptoms were 2.4 times (AOR = 2.40, 95% UI (1.45-3.99)) more likely to acquire RBD symptoms than their counterparts (Table 3).

## 4. Discussion

This study intends to determine the prevalence of self-reported RBD symptoms and its associated factors among the University of Gondar medicine and health science students in Ethiopia. It helps to design and implement screening of at-risk students for RBD to ameliorate their living standards and performance. The prevalence of RBD symptoms in the current study is 46.25% (UI: 41.26%-51.24) which is a major public health problem. The higher prevalence of RBD symptoms could be related with the competitive and demanding academic culture of the university. Even there were findings of extremely higher REM sleep behavior disorder in certain disease state individuals like in Parkinson's disease patients (42.3-66%) [13, 35], in multiple system atrophy (76.1%) [36], and in UAE (67.2%) [37]. The possible reason for the higher prevalence of these diseases might be that sleep behavior disorder is manifested in neurological diseases. There were other studies which reported a lower prevalence than our finding as seen in North Carolina (27%) [24] and the King University of Saudi Arabia (36.6%) [11]. This could be for variations in lifestyle and socioeconomic differences. To the best of our knowledge, there are insufficient published articles on the prevalence of RBD symptoms in the world as well as in Ethiopia. Because of this paucity of studies, we are not able to compare and discuss our result with others. In this study, excessive daytime sleepiness, depressive symptoms, and perceived stress were experienced in 31.07%, 34.73%, and 81.25%, respectively. Daytime sleepiness is similar with other studies [24, 38], higher than [39], and lower than [40]. Our study finding revealed a prevalence of depressive symptoms similar with [41], lower than [42], and higher than [43]. The perceived stress level in our study is higher than a study conducted in Jimma [44] and lower than a study in Pakistan [45]. The aforementioned differences might be accounted by variations in the learning environment, lifestyle, and sample size used.

In the final model, performing physical exercise, Facebook utilization immediately before going to bed, excessive daytime sleepiness, and depressive symptoms were significantly associated with self-reported RBD symptoms. The odds of acquiring RBD symptoms was 2.5 times higher in those who performed physical exercise immediately before going to bed than those who do not. This is supported by a review [46], but it is against other studies [47, 48]. The possible biological mechanism for the association of physical exercise and RBD symptoms could be due to the fact that exercise-induced body temperature rise, and accelerated cardiac activities interrupt sleep and also physical exercise prolongs non-REM sleep and shortens REM sleep phases which attributes to RBD symptoms [49]. Self-reported RBD symptoms are 1.93 times higher among students who used Facebook immediately before going to bed, which is in agreement with other studies where electronic media use interrupts sleep [50–53]. The possible reason for this association might be due to the scenes seen on Facebook, and any gestures dreamed at sleep time lead to RBD symptoms. The other link could be the contents seen in Facebook which could induce depressive symptoms and anxiety which ended up with sleep disturbances [53]. Students who experienced daytime sleepiness are more likely to develop RBD symptoms, which is supported by another study [54]. This might be because daytime sleepiness disturbs circadian rhythm, which leads to the occurrence of RBD [55–57]. However, there is a study against our findings [58]. The last we need to discuss is the association of depressive symptoms and RBD symptoms. Students who reported depressive symptoms were 2.4 times more likely to experience RBD symptoms. This is congruent with other study [59]. The probable reason for the association might be due to the fact that a person with depression can have hormonal disturbances that may disturb sleep time and hence unrestful sleep [60]. The use of antidepressants may lead to RBD even though there is no data regarding the use of antidepressants among the study participants.

## 5. Limitations of the Study

There are several limitations in this study. The self-reported nature of the RBD tool indicates the probable existence of RBD symptoms but not the real diagnosis of RBD; i.e., most participants with RBD symptoms will not have a confirmed diagnosis of RBD and also it cannot discriminate RBD and sleepwalking. The BDI-II tool is also a screening instrument for depressive symptoms, which cannot show the confirmed diagnosis of depression. Besides, recall bias and the nature of cross-sectional design cannot show a cause-effect relationship.

## 6. Conclusions

The prevalence of self-reported RBD symptoms was high in this study. Daytime sleepiness, physical exercise immediately before sleep, Facebook utilization immediately before sleep, and depressive symptoms were significantly associated with RBD symptoms. The findings of the study need the education sector to design screening strategies for depressive symptoms and daytime sleepiness for preventing RBD symptoms and hence improving their living standards.

## Figures and Tables

**Table 1:** Sociodemographic profiles of study participants in the University of Gondar, Northwest Ethiopia, 2019 (*n* = 387).

Variables	Categories	Frequency	Percent (%)
Sex	Male	271	70.03
	Female	116	29.97

Romantic relation	Yes	107	27.65
	No	280	72.35

Residence before university	Urban	226	58.40
	Rural	161	41.60

Age of respondents	20 years and below	197	50.90
	21 years and above	190	49.10

Monthly pocket money in ETB	50-400 ETB	104	26.87
	401-500 ETB	117	30.23
	501-1000 ETB	125	32.30
	1001-3800 ETB	41	10.59

Religion	Orthodox	319	82.43
	Muslim	25	6.46
	Protestant	36	9.30
	Catholic	7	1.81

Year of study	First year	149	38.50
	Second year and above	238	61.50

Field of study	Medicine	81	20.93
	Health science	306	79.07

**Table 2:** Sleep-related variables and bedtime routine activities of the study participants at the University of Gondar, Northwest Ethiopia, 2019 (*n* = 387).

Variables	Frequency	Percent (%)
Presence of bruxism		
No	339	87.60
Yes	48	12.40
Drinking coffee before sleep		
No	343	88.63
Yes	44	11.37
Drinking alcohol before sleep		
No	361	93.28
Yes	26	6.72
Smoke cigarette before sleep		
No	362	93.54
Yes	25	6.46
Physical exercise before sleep		
No	323	83.46
Yes	64	16.54
Watching TV before sleep		
No	207	53.49
Yes	180	46.51
Taking bath before sleep		
No	260	67.18
Yes	127	32.82
Using Facebook before sleep		
No	148	38.24
Yes	239	61.76
REM sleep behavior disorder score		
No	208	53.75
Yes	179	46.25
Excessive daytime sleepiness (*n* = 383)		
No	264	68.93
Yes	119	31.07
Depression (*n* = 383)		
No	250	65.27
Yes	133	34.73
Stress (*n* = 384)		
No	72	18.75
Yes	312	81.25

**Table 3:** Factors associated with self-reported symptoms of RBD among the University of Gondar medicine and health science students, Northwest Ethiopia, 2019 (*n* = 387).

Variables	RBD symptoms		COR (95% UI)	AOR (95% UI)
	No	Yes		
	Number (%)	Number (%)		
Romantic relationship				
Yes	48 (44.86)	59 (55.14)	1.64 (1.05-2.57)	1.26 (0.74-2.13)
No	160 (57.14)	120 (42.86)	1	1
Residence				
Urban	139 (61.5)	87 (38.5)		
Rural	69 (42.86)	92 (57.14)	2.13 (1.41-3.21)	1.50 (0.91-2.48)
Bruxism				
No	190 (56.05)	149 (43.95)	1	1
Yes	18 (37.50)	30 (62.50)	2.13 (1.14-3.96)	1.16 (0.53-2.54)
Drink coffee before sleep				
No	194 (56.56)	149 (43.44)	1	1
Yes	14 (31.82)	30 (68.18)	2.79 (1.43-5.45)	1.82 (0.77-4.31)
Drink alcohol before sleep				
No	201 (55.68)	160 (44.32)	1	1
Yes	7 (26.92)	19 (73.08)	3.41 (1.39-8.31)	1.64 (0.56-4.77)
Smoking cigarette				
No	203 (56.08)	159 (43.92)	1	1
Yes	5 (20.00)	20 (80.00)	5.11 (1.88-13.91)	1.67 (0.45-6.29)
Physical exercise				
No	189 (58.51)	134 (41.49)	1	1
Yes	19 (29.69)	45 (70.31)	3.34 (1.87-5.97)	2.50 (1.24-5.02)^∗∗^
Bathing before sleep				
No	150 (57.69)	110 (42.31)	1	1
Yes	58 (45.67)	69 (54.33)	1.62 (1.06-2.49)	0.99 (0.59-1.67)
Using Facebook before sleep				
No	93 (62.84)	55 (37.16)	1	1
Yes	115 (48.12)	124 (51.88)	1.82 (1.20-2.77)	1.93 (1.18-3.15)^∗∗∗^
Age in years				
<21	118 (59.90)	79 (40.10)	1	1
≥21	90 (47.37)	100 (52.63)	1.66 (1.11-2.48)	1.27 (-0.79-2.04)
Pocket money (ETB)				
50-400	43 (41.35)	61 (58.65)	3.87 (1.75-8.55)	2.29 (0.92-5.74)
401-500	62 (52.99)	55 (47.01)	2.42 (1.11-5.28)	1.73 (0.73-4.11)
501-1000	73 (58.40)	52 (41.60)	1.94 (0.89-4.23)	1.22 (0.51-2.92)
1001-3800	30 (73.17)	11 (26.83)	1	1
Excessive daytime sleepiness (n = 383)				
No	159 (60.23)	105 (39.77)	1	1
Yes	47 (39.50)	72 (60.50)	2.32 (1.49-3.61)	1.92 (1.16-3.19)^∗∗^
Depressive symptoms (n = 383)				
No	160 (64.00)	90 (36.00)	1	1
Yes	46 (34.59)	87 (65.41)	3.36 (2.16-5.23)	2.40 (1.45-3.99)^∗∗∗^
Perceived stress (n = 384)				
No	52 (72.22)	20 (27.78)	1	1
Yes	154 (49.36)	158 (50.64)	2.67 (1.52-4.67)	1.79 (0.95-3.38)
Field of study				
Medicine	53 (65.43)	28 (34.57)	1	1
Health sciences	155 (50.65)	151 (49.35)	1.84 (1.11-3.07)	0.91 (0.49-1.69)

AOR = adjusted odds ratio; COR = crude odds ratio; ETB = Ethiopian birr; ^∗∗^Significant at *p* < 0.01; ^∗∗∗^*p* < 0.001; Hosmer-Lemeshow goodness of fit (Prob > chi^2^ = 0.1181) is accepted; 1 = indicator; UI = uncertainty interval.

## Data Availability

The dataset is available from the corresponding author upon reasonable request.

## Abbreviations

AOR: Adjusted odds ratio
COR: Crude odds ratio
EPI Info: Epidemiological information
REM: Rapid eye movement
RBD: Rapid eye movement sleep behavior disorder
UI: Uncertainty interval.

## References

[1] Lee-Chiong T. (2008). *Sleep medicine: Essentials and review*.

[2] Peever J., Fuller P. M. (2016). Neuroscience: a distributed neural network controls REM sleep. *Current Biology*.

[3] Breslau N., Roth T., Rosenthal L., Andreski P. (1996). Sleep disturbance and psychiatric disorders: a longitudinal epidemiological study of young adults. *Biological Psychiatry*.

[4] Schenck C. H., Mahowald M. W. (2002). REM sleep behavior disorder: clinical, developmental, and neuroscience perspectives 16 years after its formal identification in SLEEP. *Sleep*.

[5] Sateia M. J. (2014). International classification of sleep disorders-third edition. *Chest*.

[6] Zoetmulder M., Nikolic M., Biernat H., Korbo L., Friberg L., Jennum P. (2016). Increased motor activity during REM sleep is linked with dopamine function in idiopathic REM sleep behavior disorder and Parkinson disease. *Journal of Clinical Sleep Medicine*.

[7] Vendette M., Montplaisir J., Gosselin N. (2012). Brain perfusion anomalies in rapid eye movement sleep behavior disorder with mild cognitive impairment. *Movement Disorders*.

[8] Strobel A. V., Tankisi H., Finnerup N. B. (2018). Somatosensory function is impaired in patients with idiopathic REM sleep behaviour disorder. *Sleep Medicine*.

[9] Barone D. A., Ebben M. R., Samie A., Mortara D., Krieger A. C. (2015). Autonomic dysfunction in isolated rapid eye movement sleep without atonia. *Clinical Neurophysiology*.

[10] Miyamoto T., Miyamoto M., Iwanami M. (2010). Olfactory dysfunction in idiopathic REM sleep behavior disorder. *Sleep Medicine*.

[11] Abdulghani H. M., Alrowais N. A., Bin-Saad N. S., al-Subaie N. M., Haji A. M. A., Alhaqwi A. I. (2012). Sleep disorder among medical students: relationship to their academic performance. *Medical Teacher*.

[12] Zhou J., Zhang J., Lam S. P. (2016). Mortality and its risk factors in patients with rapid eye movement sleep behavior disorder. *Sleep*.

[13] Zhang X., Sun X., Wang J., Tang L., Xie A. (2017). Prevalence of rapid eye movement sleep behavior disorder (RBD) in Parkinson’s disease: a meta and meta-regression analysis. *Neurological Sciences*.

[14] Gomutbutra P., Kanjanaratanakorn K., Tiyapun N. (2018). Prevalence and clinical characteristics of probable REM behavior disorder in Thai Parkinson’s disease patients. *Parkinson’s Disease*.

[15] Teman P. T., Tippmann-Peikert M., Silber M. H., Slocumb N. L., Auger R. R. (2009). Idiopathic rapid-eye-movement sleep disorder: associations with antidepressants, psychiatric diagnoses, and other factors, in relation to age of onset. *Sleep Medicine*.

[16] Schenck C. H., Mahowald M. W. (2001). Rapid eye movement sleep behavior disorder. *Epilepsy and Sleep*.

[17] Kang S. H., Yoon I. Y., Lee S. D., Han J. W., Kim T. H., Kim K. W. (2013). REM sleep behavior disorder in the Korean elderly population: prevalence and clinical characteristics. *Sleep*.

[18] Bixler E. O. (1979). Prevalence of sleep disorders in the Los Angeles metropolitan area. *The American Journal of Psychiatry*.

[19] Berhanu H., Mossie A., Tadesse S., Geleta D. (2018). Prevalence and associated factors of sleep quality among adults in Jimma Town, Southwest Ethiopia: a community-based cross-sectional study. *Sleep Disorders*.

[20] Lemma S., Gelaye B., Berhane Y., Worku A., Williams M. A. (2012). Sleep quality and its psychological correlates among university students in Ethiopia: a cross-sectional study. *BMC Psychiatry*.

[21] Ferber R., Kryger M. H. (1995). *Principles and practice of sleep medicine in the child*.

[22] Brown F. C., Soper B., Buboltz W. C. (2001). Prevalence of delayed sleep phase syndrome in university students. *College Student Journal*.

[23] Alqudah M., Balousha S. A. M., al-Shboul O., al-Dwairi A., Alfaqih M. A., Alzoubi K. H. (2019). Insomnia among medical and paramedical students in Jordan: impact on academic performance. *BioMed Research International*.

[24] Gaultney J. F. (2010). The prevalence of sleep disorders in college students: impact on academic performance. *Journal of American College Health*.

[25] Schlarb A. A., Kulessa D., Gulewitsch M. D. (2012). Sleep characteristics, sleep problems, and associations of self-efficacy among German university students. *Nature and Science of Sleep*.

[26] Angelone A. M., Mattei A., Sbarbati M., di Orio F. (2011). Prevalence and correlates for self-reported sleep problems among nursing students. *Journal of Preventive Medicine and Hygiene*.

[27] Sunwoo J. S., Kim Y. J., Byun J. I. (2020). Comorbid depression is associated with a negative treatment response in idiopathic REM sleep behavior disorder. *Journal of Clinical Neurology*.

[28] Barone D. A., Henchcliffe C. (2018). Rapid eye movement sleep behavior disorder and the link to alpha-synucleinopathies. *Clinical Neurophysiology*.

[29] Stiasny-Kolster K., Mayer G., Schäfer S., Möller J. C., Heinzel-Gutenbrunner M., Oertel W. H. (2007). The REM sleep behavior disorder screening questionnaire—a new diagnostic instrument. *Movement Disorders*.

[30] Beck A. T., Steer R. A., Brown G. K. (1996). *Beck depression inventory-II*.

[31] Cohen S., Kamarck T., Mermelstein R. (1994). *Perceived stress scale. Measuring stress: A guide for health and social scientists*.

[32] Johns M. W. (1991). A new method for measuring daytime sleepiness: the Epworth sleepiness scale. *Sleep*.

[33] Duko B., Erdado M., Ebrahim J. (2019). Prevalence and factors associated with depression among hospital admitted patients in South Ethiopia: cross sectional study. *BMC Research Notes*.

[34] Johns M. W. (1992). Reliability and factor analysis of the Epworth sleepiness scale. *Sleep*.

[35] Bernath O., Guilleminault C., Comella C. L., Nardine T. M., Diederich N. J., Stebbins G. T. (1999). Sleep-related violence, injury, and REM sleep behavior disorder in PD. *Neurology*.

[36] Palma J.-A., Fernandez-Cordon C., Coon E. A. (2015). Prevalence of REM sleep behavior disorder in multiple system atrophy: a multicenter study and meta-analysis. *Clinical autonomic research : official journal of the Clinical Autonomic Research Society*.

[37] Afandi O., Hawi H., Mohammed L. (2012). Sleep quality among university students in the UAE. *Gulf Medical Journal*.

[38] Whittier A., Sanchez S., Castañeda B. (2014). Eveningness chronotype, daytime sleepiness, caffeine consumption, and use of other stimulants among Peruvian university students. *Journal of Caffeine Research*.

[39] Shen Y., Meng F., Tan S. N. (2019). Excessive daytime sleepiness in medical students of Hunan province: prevalence, correlates, and its relationship with suicidal behaviors. *Journal of Affective Disorders*.

[40] Alsaggaf M., Wali S., Merdad R., Merdad L. (2016). Sleep quantity, quality, and insomnia symptoms of medical students during clinical years: relationship with stress and academic performance. *Saudi Medical Journal*.

[41] Sarokhani D., Delpisheh A., Veisani Y., Sarokhani M. T., Manesh R. E., Sayehmiri K. (2013). Prevalence of depression among university students: a systematic review and meta-analysis study. *Depression Research and Treatment*.

[42] Wahed W. Y. A., Hassan S. K. (2017). Prevalence and associated factors of stress, anxiety and depression among medical Fayoum University students. *Alexandria Journal of Medicine*.

[43] Chen L., Wang L., Qiu X. H. (2013). Depression among Chinese university students: prevalence and socio-demographic correlates. *PLoS One*.

[44] Henok Shiferaw N., Anand S., Nemera N. G. (2015). Stress and coping strategies among generic B. Sc. nursing students of Jimma University, South West Ethiopia. *International Journal of Recent Advances in Multidisciplinary Research*.

[45] Shaikh B., Kahloon A., Kazmi M. (2004). Students, stress and coping strategies: a case of Pakistani medical school. *Education for Health*.

[46] Horne J. A. (1981). The effects of exercise upon sleep: a critical review. *Biological Psychology*.

[47] Flausino N. H., da Silva Prado J. M., Queiroz S. S., Tufik S., Mello M. T. (2012). Physical exercise performed before bedtime improves the sleep pattern of healthy young good sleepers. *Psychophysiology*.

[48] Dworak M., Wiater A., Alfer D., Stephan E., Hollmann W., Strüder H. K. (2008). Increased slow wave sleep and reduced stage 2 sleep in children depending on exercise intensity. *Sleep Medicine*.

[49] Kubitz K. A., Landers D. M., Petruzzello S. J., Han M. (1996). The effects of acute and chronic exercise on sleep. A meta-analytic review. *Sports Medicine*.

[50] Das-Friebel A., Perkinson-Gloor N., Brand S. (2019). A pilot cluster-randomised study to increase sleep duration by decreasing electronic media use at night and caffeine consumption in adolescents. *Sleep Medicine*.

[51] Mamun M. A. A., Griffiths M. D. (2019). The association between Facebook addiction and depression: a pilot survey study among Bangladeshi students. *Psychiatry Research*.

[52] Levenson J. C., Shensa A., Sidani J. E., Colditz J. B., Primack B. A. (2016). The association between social media use and sleep disturbance among young adults. *Preventive Medicine*.

[53] Wolniczak I., Cáceres-DelAguila J. A., Palma-Ardiles G. (2013). Association between Facebook dependence and poor sleep quality: a study in a sample of undergraduate students in Peru. *PLoS One*.

[54] Sakaguchi K., Yagi T., Maeda A. (2014). Association of problem behavior with sleep problems and gastroesophageal reflux symptoms. *Pediatrics International*.

[55] Chang A.-M., Aeschbach D., Duffy J. F., Czeisler C. A. (2015). Evening use of light-emitting eReaders negatively affects sleep, circadian timing, and next-morning alertness. *Proceedings of the National Academy of Sciences of the United States of America*.

[56] Green A., Cohen-Zion M., Haim A., Dagan Y. (2017). Evening light exposure to computer screens disrupts human sleep, biological rhythms, and attention abilities. *Chronobiology International*.

[57] Weissová K., Škrabalová J., Skálová K. (2018). Circadian rhythms of melatonin and peripheral clock gene expression in idiopathic REM sleep behavior disorder. *Sleep Medicine*.

[58] Vukoja M., Kopitovic I., Milicic D., Maksimovic O., Pavlovic-Popovic Z., Ilic M. (2018). Sleep quality and daytime sleepiness in patients with COPD and asthma. *The Clinical Respiratory Journal*.

[59] Ghorayeb I., Yekhlef F., Chrysostome V., Balestre E., Bioulac B., Tison F. (2002). Sleep disorders and their determinants in multiple system atrophy. *Journal of Neurology, Neurosurgery, and Psychiatry*.

[60] Nutt D., Wilson S., Paterson L. (2008). Sleep disorders as core symptoms of depression. *Dialogues in Clinical Neuroscience*.

